# Appraisal of the Karnofsky Performance Status and proposal of a simple algorithmic system for its evaluation

**DOI:** 10.1186/1472-6947-13-72

**Published:** 2013-07-19

**Authors:** Dominik Péus, Nicolas Newcomb, Silvia Hofer

**Affiliations:** 1 Department of Neurosurgery, University Hospital Zurich, Zurich, Switzerland; 2 Institute of Biomedical Ethics, University of Zurich, Zurich, Switzerland; 3 Department of Oncology, University Hospital Zurich, Zurich, Switzerland

**Keywords:** Karnofsky performance status, Quality of life, Disability evaluation, Algorithms, Neoplasms, Review

## Abstract

**Background:**

For over 60 years, the Karnofsky Performance Status (KPS) has proven itself a valuable tool with which to perform measurement of and comparison between the functional statuses of individual patients. In recent decades conditions for patients have changed, and so too has the KPS undergone several adjustments since its initial development.

**Discussion:**

The most important works regarding the KPS tend to focus upon a variety of issues, including but not limited to reliability, validity and health-related quality of life. Also discussed is the question of what quantity the KPS may in fact be said to measure. The KPS is increasingly used as a prognostic factor in patient assessment. Thus, questions regarding if and how it affects survival are relevant.

In this paper, we propose an algorithm which uses a minimum of two and a maximum of three questions to facilitate an adequate and efficient evaluation of the KPS.

**Summary:**

This review honors the original intention of the discoverer and gives an overview of adaptations made in recent years. The proposed algorithm suggests specific updates with the goal of ensuring continued adequacy and expediency in the determination of the KPS.

## Background

The Karnofsky Performance Status (KPS) is a widely used method to assess the functional status of a patient. It was introduced by David A. Karnofsky and Joseph H. Burchenal in 1949 in an article originally published as a chapter of the book Evaluation of Chemotherapeutic Agents, edited by Colin M. MacLeod [1]. This book summarized the results of a symposium in New York in 1948, and it is for this reason that the original article is not listed in PubMed. Originally entitled Performance Status, the term Karnofsky Performance Status was coined at a later date, and renamed after the author of its creation.

The KPS describes a patient’s functional status as a comprehensive 11-point scale correlating to percentage values ranging from 100% (no evidence of disease, no symptoms) to 0% (death). The ECOG Performance Status (ECOG PS), an alternative status assessment, was developed by the Eastern Cooperative Oncology Group and derived from the KPS [2]. For years, the KPS and ECOG PS have been important tools in clinical practice. In clinical trials the two assessment methodologies are used as selection criteria (similar to processes for selection using age or gender) and for the stratification of subgroups in test patient cohorts. Along with disease staging in terms of tumor size, e.g. TNM, the KPS has established itself as a decision aid with relevance regardless of whether a patient is to receive either tumor-specific, or merely symptomatic treatment.

Furthermore, independent of the role it plays in treatment modality decisions, the KPS has also established itself as a salient prognostic factor in a variety of tumor entities.

Despite the prevalent role it holds in general oncology, the body of literature pertaining to the KPS scale is relatively succinct; most significant work regarding it was developed in the 1980s.

A central theme of this article is definable by the following question: “How is it possible that a subjective value assigned by clinicians within a matter of seconds, is given the same or even greater prognostic significance than many objective values?” The objective values referred to in this case being, for example, prognostic estimates based on genetic testing, elaborate staging investigations, etc. The question posed here does not intend to doubt the importance of various objective methodologies, nor their continued development, rather it seeks simply to demonstrate that the financially free and quickly detectable value, KPS, carries undeniable weight, and that due to this its critical evaluation remains a relevant issue.

## Discussion

### Questioning the adequacy and objectivity of the KPS

#### Subjectivity versus objectivity of the KPS

When evaluating the KPS questions arise regarding both its objectivity and validity. Indeed, questions of objectivity and the influence of chemotherapy on KPS scores were already engaging David A. Karnofsky in the early days of cytotoxic drug research in the 1940s. At the time he made the incisive observation that whenever a chemotherapeutic agent succeeds in relieving symptoms, this results in the subjective judgment that it is effective by the patient concerned; the treating clinician, however, must rely on objective criteria to evaluate a drug’s efficacy. In response to this, David A. Karnofsky developed criteria which even today remain valid for the assessment of chemotherapy: tumor size, laboratory parameters (e.g. anemia), length of remission, and overall survival. All of these criteria share commonalities in that they are discretely measurable and therefore objectifiable. According to Karnofsky, symptoms such as general weakness, vomiting, skin rashes and pain were to be considered subjective variables - subjective, because they were not precisely quantifiable and could be erroneously influenced by subjective experience, as well as personal factors and interests.

Not to be ignored, however, the objective assessment criteria listed above may not be taken as the only basis for patient evaluation. They fail to provide any information regarding the overall state of a patient or the extent of his or her independence or need for supportive care.

In this context, Karnofsky introduced the Performance Status, “[…] describing the patient’s ability to carry on his normal activity and work, or his need for a certain amount of custodial care, or his dependence on constant medical care in order to continue alive. These simple criteria serve a useful purpose, in our experience, in that they measure the usefulness of the patient or the burden that he represents to his family or society. […]” [1].

The percentages of the KPS describe three states (conditions): A (100–80%), B (70–50%) and C (40–0%). These states describe different levels of performance. “Functionality” and “performance” comprise the core concerns of the KPS (Table 1).

**Table 1:** Karnofsky performance status

**Condition**	**Percentage**	**Comments**
A: Able to carry on normal activity and to work. No special care is needed.	100	Normal, no complaints, no evidence of disease.
	90	Able to carry on normal activity, minor signs or symptoms of disease.
	80	Normal activity with effort, some signs or symptoms of disease.
B: Unable to work. Able to live at home, care for most personal needs. A varying degree of assistance is needed.	70	Cares for self, unable to carry on normal activity or to do active work.
	60	Requires occasional assistance, but is able to care for most of his needs.
	50	Requires considerable assistance and frequent medical care.
C: Unable to care for self. Requires equivalent of institutional or hospital care. Disease may be progressing rapidly.	40	Disabled, requires special care and assistance. [In bed more than 50% of the time].
	30	Severely disabled, hospitalization is indicated although death not imminent. [Almost completely bedfast].
	20	Hospitalization necessary, very sick, active supportive treatment necessary. [Totally bedfast and requiring extensive nursing care by professionals and/or family].
	10	Moribund, fatal processes progressing rapidly. [Comatose or barely arousable].
	0	Dead.

The unbracketed text is the original text of Karnofsky and Burchenal, 1949, while in square brackets [], the newly formulated KPS values 40% - 10% of Abernethy et al., 2005, may be found.

### Inter-observer reliability

Questions regarding the subjectivity of KPS scoring include the critical evaluation of inter-observer reliability for the methods involved in KPS evaluation. The most important studies on the KPS are from the early 1980s. In one study Yates et al. recruited clinical nurses and social workers to independently measure KPS scores in 52 hospitalized patients (Pearson product moment correlation = 0.69). The Pearson product moment correlation is a measure of the linear correlation between two variables. Additionally, an at-home KPS evaluation was performed by the social workers for the same patients. This produced a corresponding Pearson product moment correlation coefficient of 0.66 [3].

In Mor et al. 47 testers were recruited and trained to accurately assess the KPS. After 4 months, these testers had to repeatedly generate KPS score evaluations in the form of written patient descriptions. The Cronbach’s coefficient alpha was used to determine inter-observer reliability which was greater than 0.97, with a maximum of 1.00. The validity of the approach was further assessed by accompanying KPS evaluation with two additional physical function tests. These were the Katz ADL index [4], and an “overall quality of life assessment” which was conducted at the initial interview and at each subsequent contact involving an interview assessment [5]. In this manner, the predictive value of the KPS’s relationship to life expectancy in a population of terminal cancer patients was effectively demonstrated [6].

In 1984 Schag et al. had 293 cancer patients complete a questionnaire on physical and psychosocial stress [7]. Two different groups of testers (oncologists and mental health professionals) then assessed the KPS of these patients. The reliability of the KPS between different observers was measured i.a. using the Pearson product moment correlation, which was 0.89. Interestingly, however, the doctors generally attributed higher scores than the mental health professionals. In the same paper, using multiple regression analyses, seven behavioral issues were empirically identified which might assist in the improvement of the evaluation of individual KPS scores. The seven variables were weight loss or gain; decreased energy; difficulties with walking, driving or personal hygiene; and the inability to engage in normal work habits. The authors suggested that an additional questioning of the patient regarding these seven variables might facilitate a more precise determination of the patient’s KPS [7].

The authors Schag et al. speculated that the lower reliability observed by Yates et al. might be related to a greater proportion (63%) of patients with low KPS scores (KPS ≤ 70%) than was present within their own study (47%) [7]. Newer studies also suggest difficulties in the accurate determination of KPS scores for patients with reduced “performance statuses” [8,9] because KPS values between 40% and 10% indicate the need for hospitalization as a criteria (Table 1). Today there are many alternatives to avoid hospitalization, especially in a palliative setting, a fact which contrasts with the more limited medical care possibilities available in the 1940s. The Australia-modified Karnofsky Performance Status (AKPS) is an example of an attempt to address the inconsistency presented due to the original KPS’s inadequate reflection of current medical practice by reformulating the KPS criteria for values between 40% and 10% [8]: “40%, in bed more than 50% of the time; 30%, almost completely bedfast; 20%, totally bedfast and requiring extensive nursing care by professionals and/or family; 10%, comatose or barely arousable.” Abernethy et al. validated the modified KPS in 2005 as part of a randomized controlled trial with palliative patients. The aim of the study was to show that the AKPS has the same predictive value for overall survival as the original KPS in a specialized palliative setting. 26 clinical nurses were trained to determine performance scores. The KPS and the AKPS were then determined simultaneously for the 306 participants at 1600 timepoints. Although the AKPS score results correlated strongly with those derived using KPS, they proved in fact to be more accurately predictive regarding the survival of palliative patients classified under KPS condition C.

### Influence of KPS on survival

In the last thirty years various studies have demonstrated the prognostic value of the KPS, primarily for various cancers [10-13], but also for other disease entities [14]. The examination of certain of these gives the impression that the KPS itself affects survival - which of course is impossible, except for perhaps indirectly. The KPS is an artificial construct which measures the ability to function. Important for survival is not the KPS percentage score, rather it is the disease state and co-morbidities, and the impact of these two items upon the patient’s vitality. A disease may cause, for example, due to its consumptive nature and disruption of specific organs or organ systems, specific disorders which limit a patient’s independence and self-sufficiency.

The symptoms which are caused by a disease are assessed and weighted simplistically by the KPS classification according to their provocation of functional impairment. Therefore, the assessment of overall physical functionality as a predictor of overall survival is quite understandable pathophysiologically because poorer prognoses are generally associated with increasingly severe symptoms and a greater burden of disease.

The reverse conclusion, that the KPS measures the vitality of an individual patient is also fallacious. For example, a single, stable brain metastasis in the motor cortex is capable of massively limiting patient independence, even if the organism on the whole remains largely unaffected by the tumor, resulting in the protracted survival of the patient despite a severely reduced KPS. In contrast, another patient in the course of the same underlying disease may rapidly perish due to liver metastases with subsequent liver failure, despite only shortly before death having enjoyed a relatively high degree of autonomy. The relative overall survival of the second patient was much shorter, irrespective of his KPS score, due merely to the relative pathophysiological advantage of the first patient. This example illustrates that patient vitality depends on many factors other than merely the KPS, including but not limited to TNM staging, age, gender, molecular genetic markers, etc.

On the other hand, the two patients described above, who might as well have been characterized by similar TNM staging, age, gender and genetic marker profiles, help to demonstrate that the KPS offers an important additional evaluatory tool. In the words of David A. Karnofsky, “While it is important to know that subjective and objective improvement have been produced, the picture is filled out if we also know whether the patient remained flat on his back or was able to return to work.” [1]. The advantage the KPS offers is the ability to reproducibly quantify impairment.

### KPS to assess the quality of life?

The importance of the KPS as a tool for assessing quality of life is a regularly discussed topic in the relevant literature [15,16]. One important definition of health related quality of life (HRQoL) is that which has been developed by the World Health Organization [17]: “Quality of life is defined as an individual’s perception of their position in life in the context of the culture and value systems in which they live and in relation to their goals, expectations, standards and concerns. It is a broad ranging concept affected in a complex way by the person’s physical health, psychological state, level of independence, social relationships, and their relationship to salient features of their environment.” Use of the KPS as a measurement of quality of life has historically been and correctly continues to be a controversial topic as it is abundantly clear it can only capture some limited aspect of the broader concept. It simply was not designed with the explicit intent of addressing the inherently expansive set of questions posed by quality of life considerations. As Mor et al. recognized, the KPS is, when applicable, primarily useful in the assessment of the overall physical quality of life [6]. The false conclusion that the KPS is an adequate measure of the quality of life is probably derived from the fact that every loss of function or loss of independence may legitimately be perceived as carrying far-reaching, individually variable effects at physical, physiological and psycho-social levels, thereby influencing the processes of sickness. Therefore, cases where a loss of physical function fails to affect quality of life would appear to be the exception.

Unfortunately, within this context, it has become evident that adequate and accurate measurements of quality of life historically played only a minor role in controlled studies [16]. Today, quality of life is generally assessed using comprehensive evaluatory questionnaires [18].

### Differences between ECOG PS and KPS

With respect to a patient’s functional status, the Eastern Cooperative Oncology Group Performance Status (ECOG PS) [2], also called the WHO Performance Status or Scale, is an often used alternative to the KPS. The ECOG PS scale ranges from 0 (healthy, no pain) to 5 (death) (Table 2). According to the literature, major advantages of one method over the other do not seem to exist. The KPS, due to its eleven-stage classification, as compared to the six-stage ECOG PS classification, is somewhat more precise. Most notably, ECOG PS usage in the case of a poor functional status leads to inadequate over-simplification [19]. A study has shown, however, that the ECOG PS does perform somewhat better than the KPS when estimating lung cancer prognoses [10]. Other studies have demonstrated minor benefits regarding reliability in favor of the KPS [20], or, despite statistically same intra-observer reliability, better inter-observer reliability favoring the ECOG PS [21]. The differences in frequencies of use of the two methods within various oncological sub-specialties may best be explained historically. The KPS plays, for example, an important role in neuro-oncology; in contrast the ECOG PS plays a more important role in many other oncological sub-specialties. On PubMed, over the course of the last 5 years, the number of annual publications found using a general search for the term Eastern Cooperative Oncology Group Performance Status or ECOG PS has increased from 104 to 238 items; for the term Karnofsky Performance Status an increase from 208 to 270 items is observable. Thus, although prevalence falls in favor of the KPS, the annual increase in publications referring to the ECOG PS is greater.

**Table 2:** ECOG performance status

**Grade**	**ECOG**
0	Fully active, able to carry on all pre-disease performance without restriction
1	Restricted in physically strenuous activity but ambulatory and able to carry out work of a light or sedentary nature, e.g., light house work, office work
2	Ambulatory and capable of all selfcare but unable to carry out any work activities. Up and about more than 50% of waking hours
3	Capable of only limited selfcare, confined to bed or chair more than 50% of waking hours
4	Completely disabled. Cannot carry on any selfcare. Totally confined to bed or chair
5	Dead

### Proposal for a simple, efficient and goal-oriented KPS evaluation

The absence of a KPS classification scheme has been criticized by a variety of groups in the past. Keeping the significant works of Mor et al. and Schag et al. [6,7] as well as the newly developed Australia-modified Karnofsky Score [8], in mind, this paper would propose a method to facilitate the simple, efficient, and goal-oriented evaluation of the KPS in clinical practice. Three principle questions orient themselves according to the tripartite, conditional classification (A, B, and C), proposed in David A. Karnofsky’s original article (Table 1).

**Figure 1:**
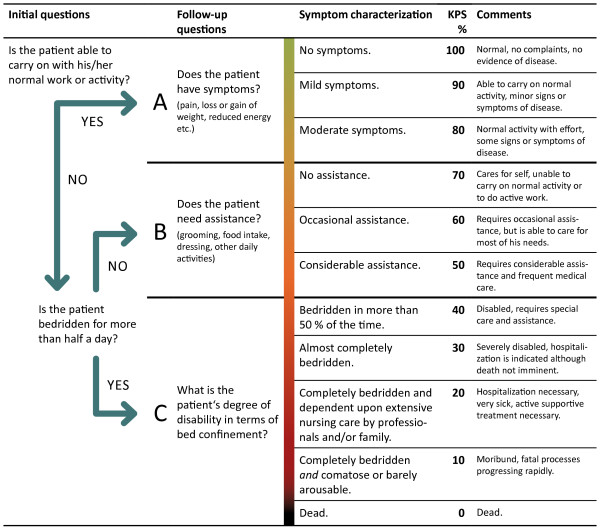
**Proposed algorithm system for the evaluation of the Karnofsky performance status.** The initial questions are answered with yes or no answers to discriminate between three statuses: A, B and C (see Table 1). The following questions further distinguish 11 derivative KPS values (100–0%). The items in round brackets () in the follow-up questions lend further suggestive clarification (Schag et al., 1984). The symptom characterization is based on the works of Karnofsky and Burchenal, 1949, and Abernethy et al., 2005 (Table 1).

The first question (1), “Is the patient able to carry on with his/her normal work or activity?”, provides stratification between state A (able to carry on normal activity and to work) and the states B and C (unable to work) (Figure 1). If question (1) is answered in the negative, question (2), “Is the patient bedridden for more than half a day?”, provides further differentiation between the states B (not able to work) and C (not capable of self-care). Subsequent to this, in each case, only one further question is necessary to determine the exact KPS percentage score. Specifically; in state A, “Does the patient have symptoms?”; in state B, “Does the patient need assistance?”; and in state C, “What is the patient”s degree of disability in terms of bed confinement?”.

The questions focus upon functional capacity. Question (2) is based on Abernethy’s findings and is intended to avoid the inadequacies of the originally proposed criteria for KPS values from 40% to 10%. In the majority of cases the proposed questions are clearly answerable and result in a consecutive, relatively discrete categorization within the 11-point scale. In everyday clinical practice the proposed algorithm system would likely prove helpful, especially to those individuals still inexperienced in performing a KPS evaluation.

Although the algorithm proposed is based on the conventional classification, the measurements which are carried out using the algorithm might not necessarily be completely consistent with previous non-algorithmically obtained measurements. A prospective study to validate and assess the algorithm would be desirable. A good method to achieve this would be through equivalence-testing between the KPS, the AKPS, and the proposed algorithm. A study consisting of three groups of examiner participants and a shared patient pool would be a sensible means to this end. The examiners would have to be instructed regarding the measurement of conventional KPS, AKPS and KPS as conducted according to the proposed algorithm, and the measurements of the three groups of examiners regarding the shared patient group would need to be conducted independently, but still as temporally consistent as possible, to avoid erroneous errors due to fluctuations in the conditions of individual patients over time.

### Unresolved issues regarding the KPS

What does a KPS score of 100% mean for an oncological patient? David A. Karnofsky describes a KPS score of 100% as “normal, no complaints, no evidence of disease” (Table 1). The first two items are relatively easily accepted and assessed. But what of the third item, “no evidence of disease”? Is the judgment to be made here meant purely to be based on external, clinical findings, irrespective of, for example, available imaging or lab diagnostics? In everyday practice, experience has demonstrated that many patients are assessed to have a KPS of 100% as long as they are independently functional and symptom-free; this is done despite their having some form of cancer and therefore demonstrable “evidence of disease”. In the literal sense a KPS of 100% must be considered a true rarity among oncology patients.

Are unwanted treatment effects (radiation, chemotherapy) reflected by KPS estimates? In everyday clinical practice, a common occurrence is that a patient’s KPS score is evidently reduced due to the initiation of treatment or intervention, at least initially. Does this carry the consequence that it is only possible to reliably evaluate KPS scores under the relatively static conditions which exist before and after treatment?

What KPS score might adequately describe, for example, a young, hemiplegic, wheelchair-dependent glioma patient, who otherwise has no symptoms and works full time? In this case, is a lower KPS score justifiable?

Who should perform a KPS evaluation? Typically, the KPS is assessed by the supervising oncologist. Research results show, however, that this evaluation could be done by the nursing staff, or even by the patient.

And what of the somewhat vague formulations of KPS comments such as, “40% or less”? Are the revisions proposed by Abernethy et al. a solution to this?

A consensus on these issues, as well as adequate instruction regarding the performance of KPS evaluation, are important factors to maximizing the comparability of KPS evaluations on separate patients over time, and to ensure the consistency of KPS measurements between clinical trials. The proposed algorithm is an attempt to standardize the process and therefore the results of KPS evaluation.

## Summary

In summary, the KPS is an artificial construct which measures a patient’s activity level using an 11-point scale. One may postulate that the objective assessment of the functional status of a patient is accessible using the KPS. Objectivity of this assessment is limited, however, by the fact that it is individuals with personal values who perform KPS evaluations, and that many patients may demonstrate rapid fluctuations regarding their self-sufficiency.

The algorithm system proposed by this work may be of use in clinical practice. It allows for the standardized and efficient assessment of the KPS score through the posing of a minimum of two, and a maximum of three, questions. A prospective study with the aim to investigate the proposed algorithm’s applicability and validity is necessary.

The KPS allows for the classification and stratification of patients whose clinical conditions are often highly complex. Along with the ECOG PS, it is the only method for stratifying patients in a vast and mixed arena of heterogeneous diseases and disabilities. That the KPS is adequately assessable in a variety of individual patients is the foundation of its applicability. It should not, however, be misinterpreted as a measure of quality of life.

The significance of the KPS as an important predictive value of overall survival must not be underestimated. The assessed factor, functionality, as a determining factor for overall survival, may depend upon other elements which are not taken into account by the conventional KPS evaluation.

All in all, body functions exist in a permanent and complex interdependency with activity, participation, personal and environmental factors. Clinically, the KPS of a patient must be evaluated within this framework thereby assessing some of the aspects of all of these factors.

## Abbreviations

KPS: Karnofsky performance status; AKPS: Australia-modified karnofsky performance status; ECOG PS: Eastern cooperative oncology group performance status; HRQoL: Health related quality of life; TNM: TNM classification of malignant tumors.

## Competing interests

The authors declare that they have no competing interests.

## Authors’ contributions

DP conception, review the current literature, drafting manuscript. NN drafting manuscript. SH supervision, drafting manuscript. All authors read and approved the final manuscript.

## Pre-publication history

The pre-publication history for this paper can be accessed here:

http://www.biomedcentral.com/1472-6947/13/72/prepub
